# Efficient Genome Editing by a Miniature CRISPR-AsCas12f1 Nuclease in *Bacillus anthracis*

**DOI:** 10.3389/fbioe.2021.825493

**Published:** 2022-01-14

**Authors:** Yanchun Wang, Shuli Sang, Xin Zhang, Haoxia Tao, Qing Guan, Chunjie Liu

**Affiliations:** State Key Laboratory of Pathogens and Biosecurity, Institute of Biotechnology, Academy of Military Medical Sciences, Beijng, China

**Keywords:** CRISPR-Cas12f, *Bacillus* anthracis, genome editing, endonuclease I-SceI, plasmid curing

## Abstract

A miniature CRISPR-Cas12f has been demonstrated to serve as an effective genome editing tool in gram negative bacteria as well as human cells. Here, we developed an alternative method to edit the genome of *Bacillus anthracis* based on the AsCas12f1 nuclease from *Acidibacillus sulfuroxidans*. When the *htrA* gene on the chromosome and the *lef* gene on the plasmid pXO1 were selected as targets, the CRISPR-AsCas12f1 system showed very high efficiency (100%). At the same time, a high efficiency was observed for large-fragment deletion. Our results also indicated that the length of the homologous arms of the donor DNA had a close relationship with the editing efficiency. Furthermore, a two-plasmid CRISPR-AsCas12f1 system was also constructed and combined with the endonuclease I-SceI for potential multi-gene modification. This represents a novel tool for mutant strain construction and gene function analyses in *B. anthracis* and other *Bacillus cereus* group bacteria.

## Introduction

Clustered regularly interspaced short palindromic repeats (CRISPRs) are part of the bacterial immune system that defends against invading viruses (Westra et al., 2014). They are made up of repeating sequences of genetic code that are interrupted by pieces of genetic code from previous invaders, which allows a cell to detect and destroy returning invaders. Based on this characteristic, CRISPR-associated (Cas) nucleases have been extensively used in genome editing in many species and an increasing number of newly discovered Cas nucleases have been explored as novel genome editing tools (Jiang and Marraffini, 2015; Manghwar et al., 2019). To date, the miniature CRISPR effectors, such as CasΦ, Cas13, and Cas14, have been developed into functional genome editors (Aquino-Jarquin, 2019; Savage, 2019; Pausch et al., 2020; Awan et al., 2021). Of these Cas nucleases, Cas12f has been shown to be superior as an editing system due to its small molecular weight and ease of cellular delivery (Awan et al., 2021).

Cas12f (also known as Cas14) is a family of relatively compact RNA-guided nucleases that were originally found in an uncultivated archaea species. It belongs to the class 2 type V-F CRISPR-associated effector nuclease family. Most Cas12f proteins are approximately 400–700 amino acids in length, include a single RuvC nuclease domain, and are known as miniature Cas proteins (Takeda et al., 2021; Xiao et al., 2021). Cas12f was discovered by mining a database of microbial genomes and metagenomes, and Cas12f was shown to bind and cleave a targeted ssDNA sequence using a specific gRNA sequence. However in this work, they did not determine if Cas12f was able to target dsDNA both *in vivo* and *in vitro* (Harrington et al., 2018). Next, Karvelis et al. confirmed that Cas12f nucleases recognize and cleave dsDNA in a TTTR (where R is A or G) PAM-dependent manner and have the potential to be harnessed as programmable nucleases for genome editing (Karvelis et al., 2020). Currently, Cas12f has been demonstrated to serve as an effective genome editing tool in bacteria as well as human cells. Okano et al. showed that the Un1Cas12f1 (529 aa) from an uncultured archaeon (Un1) could modify the *Escherichia coli* genome with high efficiency (50–100%) (Okano et al., 2021). Kim et al. showed that an optimized CRISPR/Un1Cas12f1 system enabled efficient and specific genome editing in human cells, with efficiency and specificity similar to that of SpCas9 and AsCas12a (Kim et al., 2021). Wu et al. also showed that AsCas12f1 from *Acidibacillus sulfuroxidans* could serve as an effective genome editing tool in both bacteria and human cells. Moreover, this AsCas12f1 system could be delivered by various delivery methods, including plasmid, ribonucleoprotein and adeno-associated virus (Wu et al., 2021). At the same time, Xu et al. developed a miniature CRISPR system with Cas12f mutants, named CasMINI, which enabled robust gene editing and base editing in mammalian cells (Xu et al., 2021). Bigelyte et al. showed that SpCas12f1 functioned in both plant and human cells to produce targeted modifications (Bigelyte et al., 2021). All of these works provide sufficient evidence that Cas12f can be developed a useful tool for broad genome engineering applications.

In a previous study, we developed a genome-editing protocol for *B. cereus* group strains based on the CRISPR-Cas9 system (Wang et al., 2019). Both large fragment deletion and precise point mutations could be achieved efficiently. Unfortunately, the large size of SpCas9 (1368 amino acids) is a nonnegligible problem, and this increases the size of any genome editing plasmid using SpCas9. Like many bacteria, the transformation efficiency of *B. anthracis* decreases with the increasing of plasmid size, and this also is a significant problem in many poorly transformable strains (Ohse et al., 1995; Turgeon et al., 2006). Alternative, miniature Cas nucleases would be a better choice in *B. anthracis* and other similar strains.

In the present study, the feasibility of CRISPR-Cas12f in *B. anthracis* was determined. When the *htrA* gene on the *B. anthracis* chromosome and the *lef* gene on the plasmid pXO1 were selected as targets, 100% modification rates were achieved in these experiments. Then the effect of homologous arm length on editing efficiency also was explored by comparative analysis of the results using plasmids with different lengths of homologous arms. At the same time a two-plasmid CRISPR-AsCas12f1 system was also constructed and combined with endonuclease I-*SceI* for potential multi-gene modification.

Taken together, an efficient genome editing protocol for *B. anthracis* was developed based on the CRISPR-AsCas12f1 system. This protocol will be a useful tool for mutant strain construction and gene function analyses in *B. anthracis*.

## Materials and Methods

### Bacterial Strains, Plasmids, and Growth Conditions

All bacterial strains and plasmids used in this study are listed in Table 1. *Escherichia coli* Top 10 cells were used as a cloning host, and *E. coli* SCS110 was used to prepare unmethylated plasmids. *E*. *coli* was grown aerobically in LB medium at 37°C, while *B. anthracis* was grown in BHIG medium (brain heart infusion broth with the addition of 0.5% glycerol). Kanamycin (50 μg/ml for *E. coli*, 30 μg/ml for *B. anthracis*) or erythromycin (400 μg/ml for *E. coli*, 5 μg/ml for *B. anthracis*) was added to the media at appropriate final concentrations as needed.

**TABLE 1:** Plasmids and strains used in this study.

Plasmids and strains	Relevant characteristics	Source
Plasmids		
pJOE8999	Rep pE194 (Ts), Kan^r^, Pman-cas9, PvanP*-sgRNA9, shuttle vector	Altenbuchner, (2016)
pJOE-Cas12f1	Rep pE194 (Ts), Kan^r^, Pman-AsCas12f1, PvanP*-sgRNA12, shuttle vector	This study
pJOE-Cas12f1-htrA-1	pJOE-Cas12f1 with sgRNA1-htrA and homologous arms of *htrA* from *B. anthracis* A16R	This study
pJOE-Cas12f1-htrA-1	pJOE-Cas12f1 with sgRNA2-htrA and homologous arms of *htrA* from *B. anthracis* A16R	This study
pJOE-Cas12f1-lef-1	pJOE-Cas12f1 with sgRNA1-lef and homologous arms of *lef* from *B. anthracis* A16R	This study
pJOE-Cas12f1-lef-2	pJOE-Cas12f1 with sgRNA2-lef and homologous arms of *lef* from *B. anthracis* A16R	This study
pJOE-Lam03	pJOE8999 with Cas9 and homologous arms of lam03 from *B. anthracis* A16R	Wang et al. (2019)
pJOE-Cas12f1-lam03-400	pJOE-Cas12f1 with sgRNA and 400 bp homologous arms of prophage lambdaBa03 from *B. anthracis* A16R	This study
pJOE-Cas12f1-lam03-800	pJOE-Cas12f1 with sgRNA and 800 bp homologous arms of prophage lambdaBa03 from *B. anthracis* A16R	This study
pHY304	shuttle vector, resource of *ermAM*, Erm^r^	Jeng et al. (2003)
pSS4332	shuttle vector, expressing endonuclease I-SceI	Cybulski et al. (2009)
pJOE-mScarlet	Deriving from pJOE8999, from 1 to 2844 base pairs, carrying mScarlet coded sequence without promoter	This study
pCas12f1-SceI-E	Rep pE194 (Ts), Kan^r^, Pman-AsCas12f1, PxylA-I-SceI, Erm^R^, shuttle vector	This study
pSS-FD	modified skeleton of pSS4332, PvanP*-sgRNA12, two I-SceI sites, shuttle vector	This study
pSS-FD-htrA	pSS-FD with sgRNA-lef and homologous arms of htrA from *B. anthracis* A16R	This study
*B. anthracis* strains		
*B. anthracis* A16R	pXO1+pXO2–, China vaccine strain, host for genome editing	This laboratory
*B.anthracis* A16R*ΔhtrA*	*B.anthracis* A16R with *htrA* gene deletion	This study
*B.anthracis* A16R*Δlef*	*B.anthracis* A16R with *lef* gene deletion	This study
*B.anthracis* A16R*Δlam03*	*B.anthraci*s A16R excision prophage lambdaBa03	This study
*E. coli* strains		
DH5α	Cloning strain	CWBIO, China
SCS110	*dam*–/*dcm*– strain used to produce unmethylated plasmid	Transgen, China

### Editing Plasmid Construction

All constructed plasmids used in this work are shown in Table 1, and PCR primers and the N20 sequences for each PAM sequence (TTTG) are listed in Table 2. All the sequence information of synthetic DNA fragments is supplied in supplementary information (Supplementary Table S1). *B. anthracis*-codon-optimized AsCas12f1 from *A. sulfuroxidans* with the *B. subtilis* mannose manP promoter (PmanP) and the sgRNA_V1 fragment designed according to a reference were ordered from General Biosystems (China). To introduce the CRISPR-AsCas12f1 system into a plasmid, the Cas9 cassette of plasmid pJOE8999 was replaced by this synthetic AsCas12f1 cassette, and the *EcoR*I-*Xba*I fragment of the resulting plasmid was replaced with the synthetic sgRNA sgRNA_V1 fragment. This plasmid was designated pJOE-Cas12f1.

**TABLE 2:** Primers used in this study.

Name	Sequence (5′→3′)	Purpose
UtrAF	ACG​CGT​CGA​CGA​CTA​TAG​TTT​TGG​C	PCR of homology arms for *htrA* deletion
UtrAR	TTT​GGT​CTC​GTA​AAC​TCG​GAA​TAA​AAG​AAA​GTC​TC	—
DtrAF	TTT​GGT​CTC​GTT​TAC​TTC​CCC​TCT​CTG	—
DtrAR	CTA​GCT​AGC​TCG​AAG​CAG​AAG​ACG	—
sg-htrAF1	GAA​CGT​TAA​ATA​ACG​CAC​CAC​CAC	space sequence 1 of *htrA*
sg-htrAR1	GGC​CGT​GGT​GGT​GCG​TTA​TTT​AAC	—
sg-htrAF2	GAA​CCA​TCT​ACC​TTC​TTG​CCA​TCA	space sequence 2 of *htrA*
sg-htrAR2	GGC​CTG​ATG​GCA​AGA​AGG​TAG​ATG	—
htrAiF	GAA​ACC​ATA​TAC​GAT​GTA​CGT​TCT​GG	—
htrAiR	AAG​ATG​AAA​GAA​GAT​TAC​GTG​AAA​TTG	PCR of *htrA* deletion identification
UefF	ACG​CGT​CGA​CAG​ATG​TGG​TGG​GCA​AG	PCR of homology arms for *lef* deletion
UlefR	CGG​GAT​CCG​TAA​TGT​ATT​AAA​AAT​TTT​CAA​ATG	—
DlefF	CGG​GAT​CCA​TTT​AAT​CTC​TCC​TTT​TTT​ATA​AG	—
DlefR	CTA​GCT​AGC​AAA​TCA​ATG​CGT​AAA​TTG​ACC	—
sg-lefF1	GAA​CGC​ACT​ACT​TTC​GCA​TCA​ATC	space sequence 1 of *lef*
sg-lefR1	GGC​CGA​TTG​ATG​CGA​AAG​TAG​TGC	—
sg-lefF2	GAA​CGC​TCA​ATA​GGA​ATC​TGC​AGC	space sequence 2 of *lef*
sg-lefR2	GGC​CGC​TGC​AGA​TTC​CTA​TTG​AGC	—
lefiF	GAA​ATG​GTC​AGC​ACC​GCC​AGA​AG	PCR of lef deletion identification
lefiR	TGT​GTC​TAA​TGT​AGC​AGA​TAC​ATC​TAG	—
lam03-800F	ACG​CGT​CGA​CGG​AGA​ATT​TCT​TTG​AAG	PCR of 800 bp homology arms for lambdaBa03 excision
lam03-800R	GCT​CTA​GAA​GTT​GGT​GCT​CCA​ACA​TTC	—
lam03-400F	ACG​CGT​CGA​CGG​TAG​CCC​CTT​CCA​TGA	PCR of 400 bp homology arms for lambdaBa03 excision
lam03-400R	GCT​CTA​GAA​ACT​GAG​CGT​ATC​GGT​GA	—
sg-lam03F	GAA​CCT​GAC​GAG​CCT​AAC​CCA​CGA	space sequence of lambdaBa03
sg-lam03R	GGC​CTC​GTG​GGT​TAG​GCT​CGT​CAG	—
lam03iF	CCT​GGG​ATT​GAT​GAT​ACG​ATG​G	PCR of lambdaBa03 excision identification
lam03iR	GAA​GCA​ATC​GCT​CCA​GAA​ATC​G	—
htrA-400F	ACG​CGT​CGA​CTA​CTC​CTA​ATT​GTG​CCC	PCR of 400 bp homology arms for *htrA*
htrA-400R	TGC​TCT​AGA​GAA​CTT​CTC​GTT​TAT​TTA​ATG	—
htrA-200F	ACG​CGT​CGA​CTT​GCT​TTT​GAA​ACC​ATA​TAC	PCR of 200 bp homology arms for *htrA*
htrA-200R	TGC​TCT​AGA​CGG​AAC​GTA​TTG​TGT​GCT​TC	—
htrA-100F	ACG​CGT​CGA​CAG​TTG​GGT​TTT​CAA​TTG​TC	PCR of 100 bp homology arms for *htrA*
htrA-100R	TGC​TCT​AGA​TAA​GCG​TAT​TTT​TTT​AAT​TGG	—
htrA-50F	ACG​CGT​CGA​CCC​GAT​AAA​GAA​AGT​CTC	PCR of 50 bp homology arms for *htrA*
htrA-50R	TGC​TCT​AGA​AAA​ACG​TGA​CTA​TAC​TGA​A	—
rSceIF	TTT​CCT​TTT​TGC​GTG​TGA​TGC​GCT​AAT​AAC​ATA​TAA​ACA​GCC​AGT​TG	PCR of PxylA-I-SceI cassette
rSceIR	ATA​TTT​TAG​ATG​AAG​ATT​ATT​TCT​TAA​TCA​AAA​AAC​CCC​TCA​AGA​CCC​G	—
ErmF	AAC​TGC​AGA​CAA​ATC​ACT​TAT​CAC​AAA​TC	PCR of*ermAM* cassette from pHY304
ErmR	CCG​CTC​GAG​CCT​CTT​TAG​CTC​CTT​GGA​AGC	—

To explore the feasibility of CRISPR-Cas12f system in *B. anthracis*, the *htrA* gene on the *B. anthracis* chromosome and the *lef* gene on the plasmid pXO1 were selected as targets. The 800-bp upstream and downstream regions of these target genes were amplified using *B. anthracis* A16R genomic DNA as a template and inserting it into the corresponding *Sal*I and *Xba*I sites of pJOE-Cas12f1. For the *htrA* gene, primers UhtrAF/UhtrAR and DhtrAF/DhtrAR were used, respectively. Then the small double stranded target spacer, annealed with the two complementary oligonucleotides (sg-htrAF1/sg-htrAR1or sghtrAF2/sg-htrAR2), was inserted in the location between the two *Bsa*I sites of the plasmid to obtain gene specific genome editing plasmids. In this work, two sgRNAs with different target spacers on each gene were designed and tested respectively in subsequent experiments. In the same manner, plasmids for *lef* gene deletion were constructed accordingly.

To test the effect of the length of homologous arms on the editing efficiency, a series of *htrA* specific genome editing plasmids with varying lengths (50, 100, 200, or 400 bp) of upstream and downstream regions were constructed using a similar method and also were used to delete the *htrA* gene.

To explore the feasibility of large chromosomal fragment deletions using the CRISPR-Cas12f system in *B. anthracis*, the prophage lambdaBa03 (∼16.8 kb) was selected as the target. The plasmids for lambdaBa03 deletion were constructed accordingly in the similar way. The upstream and downstream regions of lambdaBa03 were amplified using plasmid pJOE-lam03 constructed previously as template (Wang et al., 2019). In this part, two different lengths (400 bp and 800 pb) upstream and downstream regions were designed and tested the editing efficiency.

### Genome Editing With a Single Plasmid

The transformation and selection of competent cells was performed as described previously (Wang et al., 2019). pJOE-cas12f1 series plasmids were introduced by electroporation of *B. anthracis* A16R, and transformants were selected at 30°C on BHIG medium containing kanamycin. Single colonies were transferred to liquid media (with 25 μg/ml kanamycin) and incubated with shaking for 3 h at 37°C. Mannose (final concentration, 0.4%) was added to induce the expression of the Cas12f1 protein. After 3 h of cultivation, serial dilutions of this culture were plated on LB agar with 25 μg/ml kanamycin and 0.4% mannose and then incubated at 37°C overnight. Transformants were identified by colony PCR and DNA sequencing. For PCR tests, the *B. anthracis* A16R strain was used as a negative control.

### Inducible Promoter Screening

All alternative promoter fragments and mScarlet coding sequence were designed according to reference or sequence information from the NCBI database and ordered from GeneralBio Co. (China). To construct the promoter screening plasmid, the plasmid pJOE8999 was digested with *BsrG*I and *EcoR*I to release the backbone of the plasmid (from 1 to 2844 bp). Then the synthetic coding region of the mScarlet coding sequence with applicable restriction sites was ligated with this fragment to construct plasmid pJOE-mScarlet (Bindels et al., 2017). Next, alternative promoters, the bacitracin-inducible promoter (Toymentseva et al., 2012), cumate-inducible promoter (Seo and Schmidt-Dannert, 2019), or xylose-inducible promoter from *Bacillus megaterium* and *Bacillus subtilis*, were inserted upstream of the mScarlet coding sequence and the resulting plasmids were introduced into the *B. anthracis* A16R strain and selected at 30°C on BHIG medium containing kanamycin.

For comparison of the mScarlet expression levels of *B. anthracis* A16R harboring different recombinant plasmid, 5 ml of LB cultures were grown to an OD600 nm = 0.6–1.0, then the suitable inducers were added to induce target protein expression. Cultures without inducers were used as a negative control. After 10 hours of induction, the OD600 nm and fluorescence intensity of cultures (excitation at 569 nm and emission at 594 nm) were measured with a SpectraMax®i3x.

### Two-Plasmid System Editing Plasmid Construction

To cure CRISPR plasmids as soon as possible, a two-plasmid system based on CRISPR-Cas12f was also constructed. At first, the I-*Sce*I cassette (containing the xylose induced promoter and the coding region of I-*Sce*I) was inserted at a location between the *Xba*I and *EcoR*I sites in plasmid pJOE-Cas12f1 by Gibson assembly. The resulting plasmid was digested with *Xho*I and *Pst*I to remove the kanamycin resistance cassette, then the erythromycin resistance cassette amplified from pHY304 was cloned into this plasmid to replace the kanamycin resistance cassette. The resulting plasmid was designated pCas12f1-SceI-E.

At the same time, the I-*Sce*I coding region of the modified plasmid pSS4332 (a point mutation was introduced to destroy the *Bsa*I site in *repB* of this plasmid) was replaced by a synthetic DNA fragment, including the sgRNA_V1 cassette, restriction sites for homologous arm insertion, and two I-*Sce*I sites on both sides, to construct plasmid pSS-FD. Then the homologous arms and the target spacer of the *htrA* gene were inserted into the plasmid pSS-FD successively using the previously described method. The resulting plasmid was designated pSS-FD-htrA and used for genome editing.

### Genome Editing With Two Plasmids

For the two-plasmid system, *B. anthracis* A16R competent cells harboring pCas12f1-SceI plasmid were prepared using previously described methods, and then the pSS-FD-htrA plasmid was introduced by electroporation. Genome editing was triggered by inducing AsCas12f1 expression. Briefly, a strain harboring pCas12f1-SceI and pSS-FD-thrA was transferred to liquid media (with 25 μg/ml kanamycin and 5 μg/ml erythromycin), and incubated with shaking for 3 h at 37°C. Mannose (final concentration, 0.4%) was added to induce the expression of the Cas12f1 protein. After cultivation for 3 h, serial dilutions of this culture were plated on LB agar with erythromycin and 0.4% mannose and cells were incubated at 37°C overnight. Transformants were identified by colony PCR and the *B. anthracis* A16R strain was used as a negative control.

### Plasmid Curing

To cure the pSS-FD-htrA plasmid, an edited colony harboring both pCas12F and pSS-FD-htrA was transferred to liquid BHIG media (with 5 μg/ml erythromycin), and incubated with shaking for 3 h at 37°C. Xylose (final concentration, 0.4%, w/v) was added to induce the expression of the endonuclease I-*SceI* and the culture without xylose was used as negative control. After cultivation for 3 h, the culture was diluted and spread onto BHIG plates with 5 μg/ml erythromycin and 0.4% xylose, and these plates were incubated at 37°C for 16–20 h. The curing of plasmid of pSS-FD-htrA was confirmed by determining the colony sensitivity to kanamycin (25 μg/ml).

## Results

### Establishment and Improvement of the CRISPR-AsCas12f1 Genome Editing System

**FIGURE 1:**
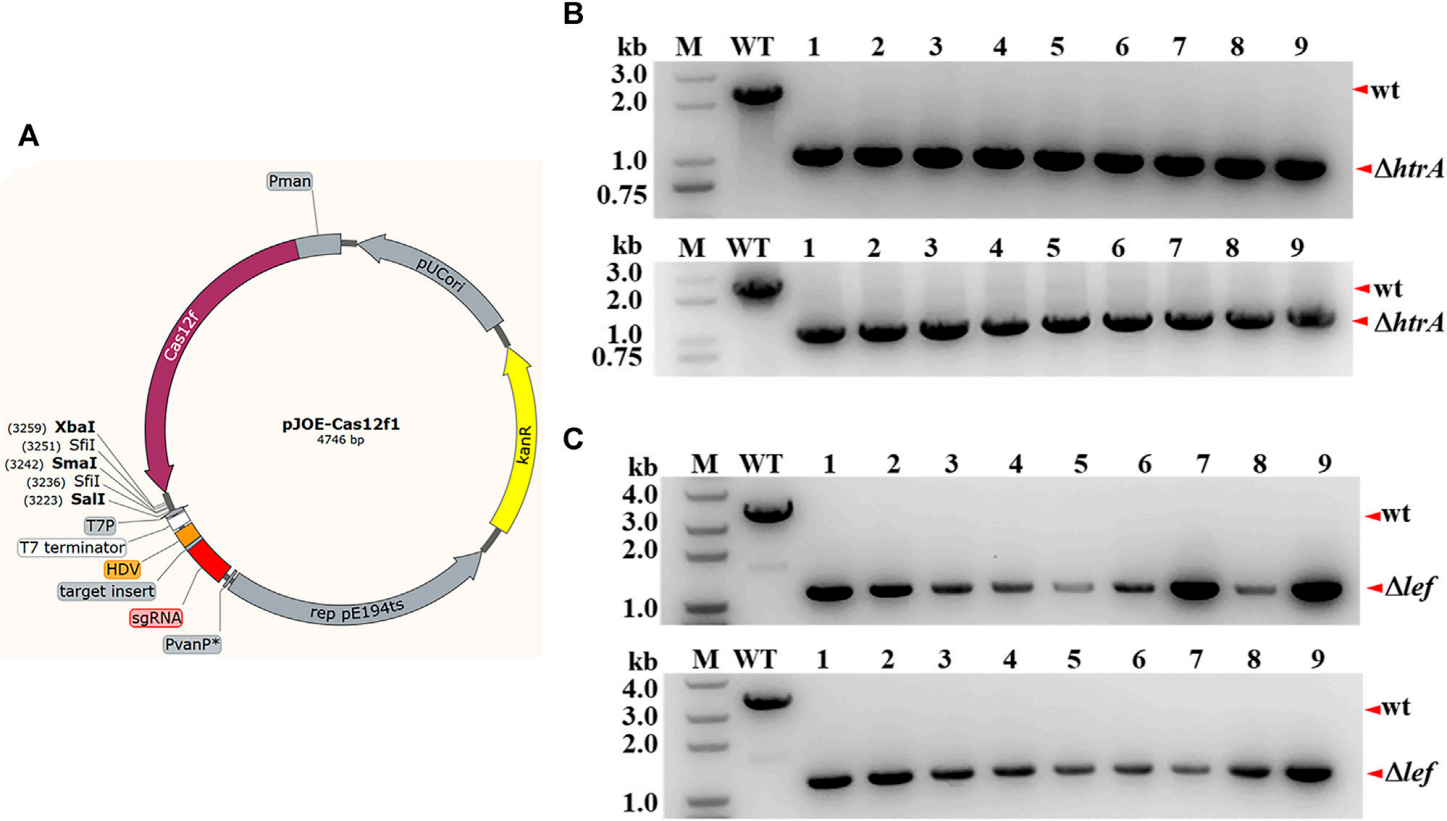
Gene deletion *via* the CRISPR-CRISPR-AsCas12f1 system in *B. anthracis*. **(A)** Physical map of plasmid pJOE-cas12f1. Pman, PmanP promoter; PvanP*, semisynthetic promoter PvanP*. The cloning sites and insertion site of the spacer sequence (*BsaI* restriction sites) are also labeled in the map. **(B)** PCR verification of *htrA* deletion in *B. anthracis* A16R. The *htrA* gene locus of the randomly selected colonies carrying the editing plasmids was amplified, and the PCR products were verified through agarose gel analysis. M, DNA marker; WT, control with *B.anthracis* A16R total DNA as the template; “wt” and “*ΔhtrA*”, wild-type band and the *htrA*-deleted band, respectively. The correct fragment in the mutant strain was approximately 1.0 kb (lanes 1–9) while in A16R this was 2.3 kb (lane WT). **(C)** PCR verification of *lef* deletion in *B. anthracis* A16R. “wt” and “*Δlef*”, wild-type band and the *lef*-deleted band, respectively. The correct fragment in the mutant strain was approximately 1.2 kb (lanes 1–9) while in A16R this was 3.6 kb (lane WT).

For demonstration of the feasibility of the CRISPR-Cas12f system in *B. anthracis*, the plasmid pJOE-AsCas12f1 was constructed and employed for genome editing (Figure 1A). First, as described in Materials and methods section, the plasmid including donor DNA, gene-specific sgRNA, and AsCas12f1 cassettes was electroporated into *B. anthracis* A16R. Then the genome editing events were performed as described previously (Wang et al., 2019). The *htrA* gene on the *B. anthracis* chromosome and the *lef* gene on the plasmid pXO1 were selected as targets, and alternative mutants were detected by colony PCR. For the *htrA* gene, compared to the 2.3 kb amplicon from the parental strain A16R, the 1.0 kb PCR products from all randomly selected colonies (9/9 and 9/9 for each target spacer) showed that *htrA* (1242 bp) had been deleted successfully (Figure 1B). A similar result was also seen for *lef* deletion (Figure 1C). These results confirmed the predominant feasibility of AsCas12f1 for *B. anthracis* genome editing.

**FIGURE 2:**
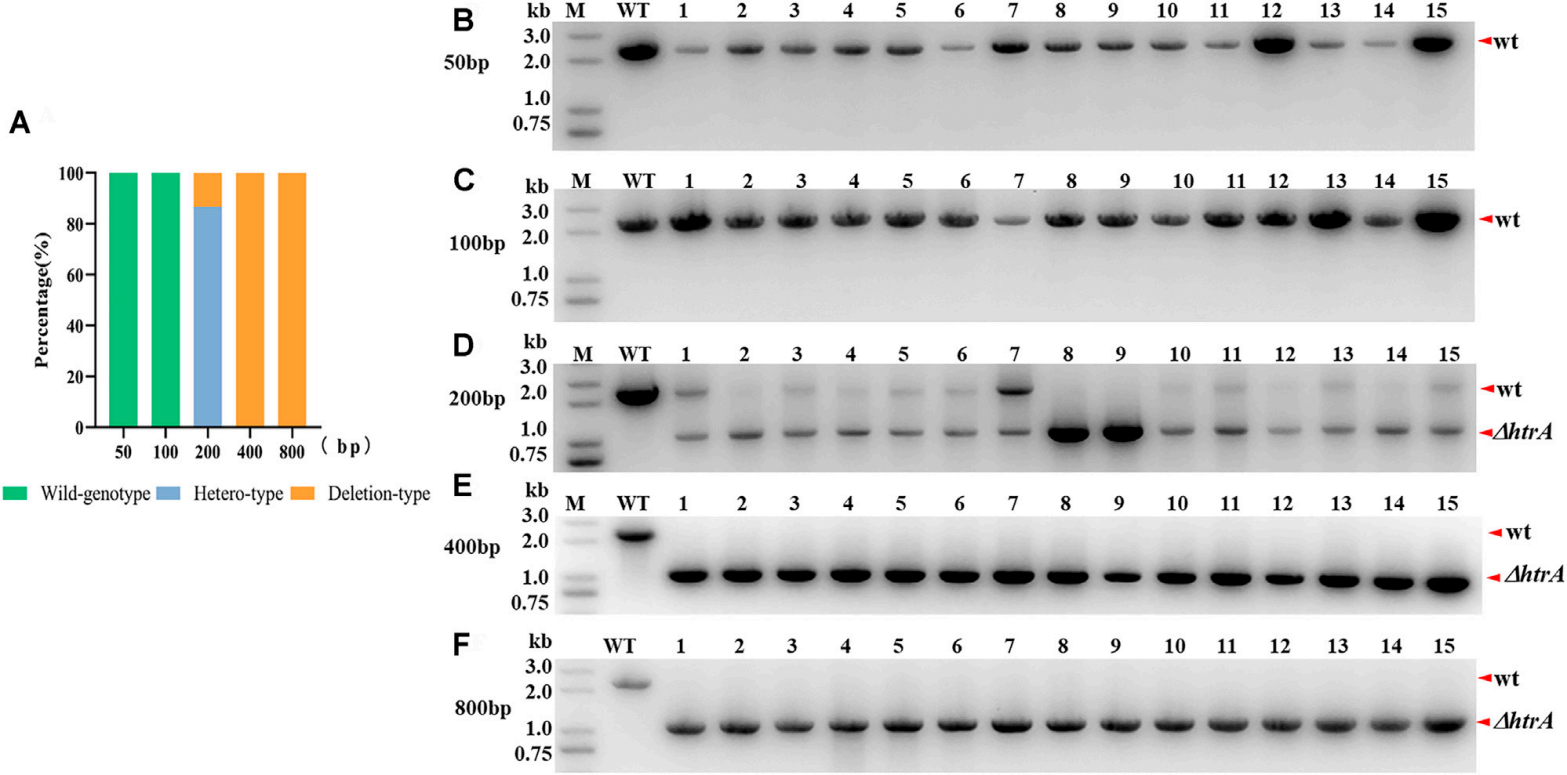
Effect of homologous arm length on gene deletion efficiency. **(A)** Comparison analysis of the genome editing efficiencies of plasmids with different length homologous arms. Hetero-type colonies showed bands for both wild type and *htrA* gene deletion. **(B–F)** PCR validation of *htrA* gene deletion in *B. anthracis* A16R using editing plasmids with different length homologous arms (50, 100, 200, 400, and 800 bp). M, DNA marker; WT, *B. anthracis* A16R total DNA as a template; wt, wild-type band; and Δ*htrA*, *htrA-*deleted band. For each plasmid group, 15 colonies were selected to validate by PCR and agarose gel analysis.

To study the relationship between the editing efficiency and the length of homologous arms, four other editing plasmids with different lengths of homologous arms (50, 100, 200, and 400 bp) were constructed. The results of *htrA* gene deletion indicated that the length of the homologous arms was closely related to the editing efficiency (Figure 2A). PCR tests indicated that when the lengths of the homologous arms were 50 bp or 100 bp, none of the 30 randomly selection colonies showed *htrA* gene deletion bands and only wild type amplification bands (Figures 2B,C). When the length of the homologous arms was 200 bp, only 2 of the 15 randomly selection colonies exhibited the *htrA* gene deletion band while most colonies (13/15) were heterozygous (two bands amplification, Figure 2D). When the length of the homologous arms was 400 or 800 bp, 100% of randomly selection colonies (15/15 and 15/15, Figures 2E,F) showed that *htrA* had been completely deleted (pure deletion type).

**FIGURE 3:**
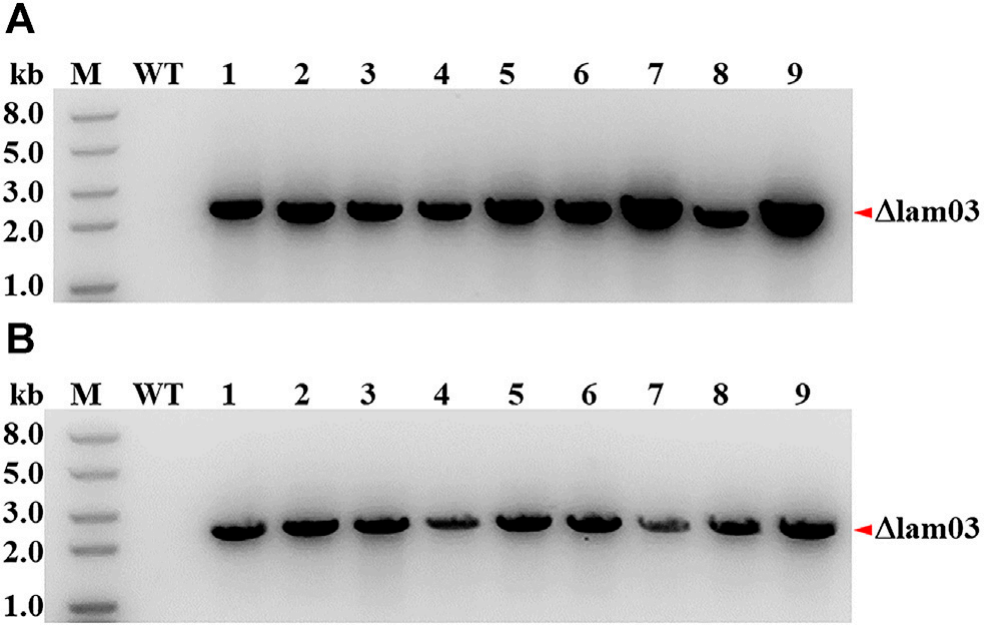
Large-fragment deletion *via* the CRISPR-CRISPR-AsCas12f1 system in B. anthracis. PCR verification of lambdaBa03 excision in *B. anthracis* A16R using editing plasmids with 800 bp **(A)** and 400 bp **(B)** homologous arms. The correct fragment amplified by the lamiF/lamiR primers in the lambdaBa03 excised strain was approximately 2.2 kb (lanes 1–9), while the region in the control strain A16R (>19 kb) exceeded the maximum amplification size under same PCR conditions. M, DNA marker; WT, control with *B.anthracis* A16R total DNA as the template; “*Δlam03*”, the lambdaBa03 excised band.

To test the availability of the CRISPR-cas12f system for longer DNA fragment deletion, the prophage lambdaBa03 was excised by similar method. 9 randomly selection colonies were screened *via* PCR. The region amplified by the lamiF/lamiR primers in the control strain A16R was > 19 kb in length, which exceeded the maximum amplification size under our PCR conditions. However, When the length of the homologous arms was 800 bp, all randomly selected colonies (9/9) showed the expected 2.2 kb amplicon, indicating that the prophage lambdaBa03 had been excised successfully (Figure 3A). At the same time, despite the substantially reduced size of the homologous arms, the 100% editing efficiency (9/9) was also seen when the 400 bp homologous arms were employed (Figure 3B).

### Two-plasmid-based CRISPR-Cas12f System and Plasmid Curing

**FIGURE 4:**
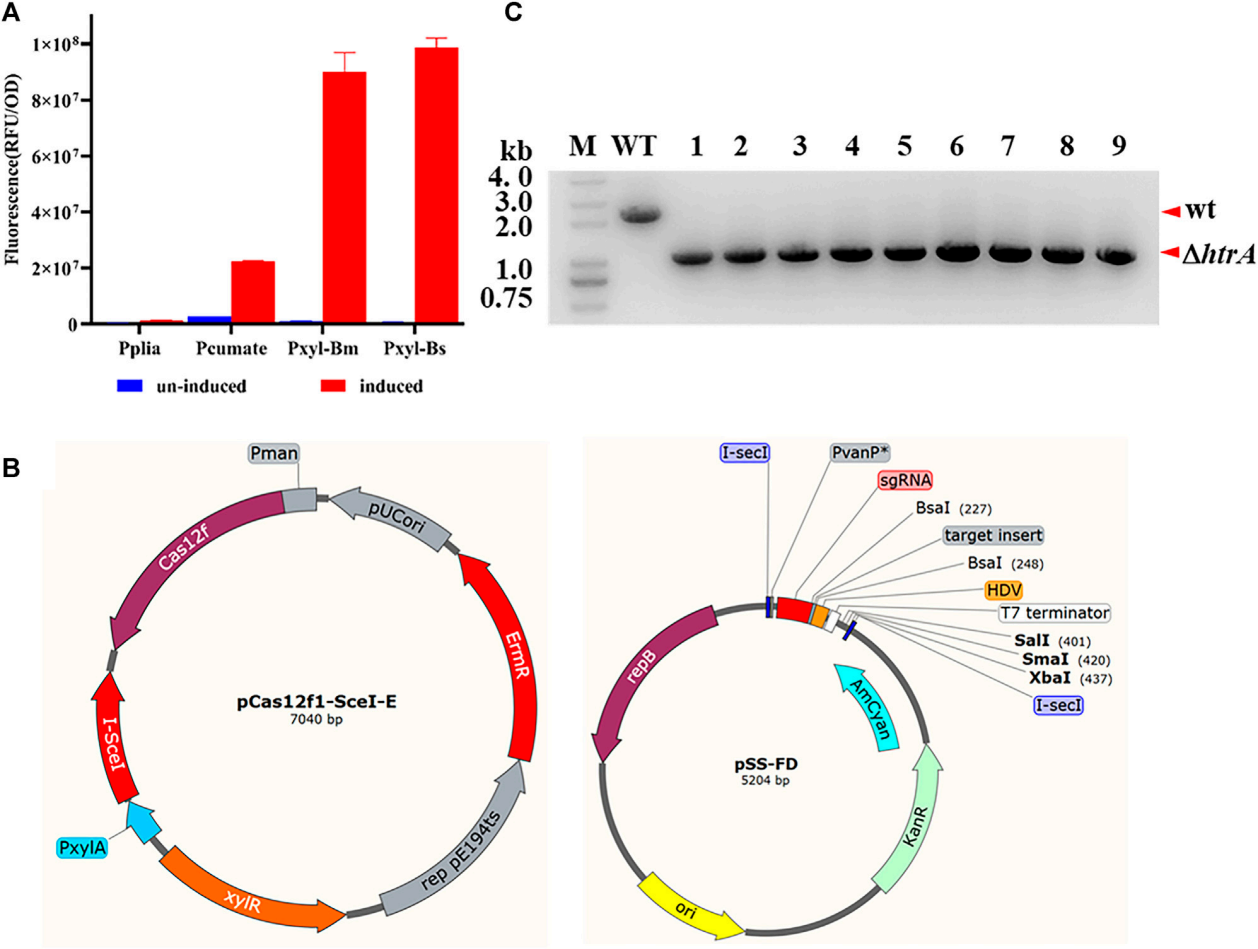
Gene deletion *via* a two-plasmid CRISPR-CRISPR-AsCas12f1 system in *B. anthracis*. **(A)** Induced promoter screening using mScarlet as a reporter molecule. **(B)** Physical map of the two-plasmid system vectors. pCas12f1-SceI-E, the plasmid co-expressed AsCas12f1 and I-*SceI* endonuclease I-*SceI* (under the control of the PxylA promoter); pSS-FD, for sgRNA expression and homologous arm supply. **(C)** PCR verification of *htrA* deletion *via* the two-plasmid system. M, DNA marker; WT, *B. anthracis* A16R total DNA as a template; wt, wild-type band; and Δ*htrA*, *htrA-*deleted band. The correct fragment in the mutant strain was approximately 1.0 kb (lanes 1–9) while in A16R this was 2.3 kb (lane WT).

To strictly induce the expression of the endonuclease I-*SceI*, four different promoters were selected to drive the expression of the report protein mScarlet. The results demonstrated that the PxylA promoter from *B. subtilis* could be strictly induced and the expression level of the report gene was relatively high (Figure 4A). Based on this result, a plasmid co-expressing AsCas12f1 and the endonuclease I-*SceI* under the control of the PxylA promoter was constructed as show in Figure 4B. Combined with another plasmid pSS-FD carrying the sgRNA cassette, this two-plasmid-based CRISPR-Cas12f system for *B. anthracis* was successfully established.

To demonstrate the feasibility of this two-plasmid system, the *htrA* gene was selected as a target once again and the editing efficiency was tested. As shown in Figure 4C, all nine randomly selected colonies (9/9) showed that *htrA* (1242 bp) had been deleted in *B. anthracis* A16R. At the same time, the result of plasmid curing tests indicated that a portion of the recombinant colonies (12/50, 6/50 and 17/50, three independent experiments) had lost kanamycin resistance and the plasmid pSS-FD-htrA had been partially eliminated after endonuclease I-*SceI* expression was induced by xylose. While no kanamycin sensitive colony was gained in the non-inducible conditions after passage once.

## Discussion

In this work, the CRISPR-Cas12f system was successfully utilized for genome editing in *B. anthracis*. The efficiency was similar to the CRISPR-SpCas9 system based on our previously reported results (Wang et al., 2019). Either the *htrA* gene on the *B. anthracis* chromosome or the *lef* gene on the plasmid pXO1 was deleted, and deletion mutants achieved rates of 100% after one round of induction and selection in these experiments.

At the same time, a high efficiency was seen for large-fragment deletion when prophage lambdaBa03 was selected as target. These results indicated that the CRISPR-12f system was a high-efficiency genetic operation tool in *B. anthracis*. Moreover, the protocol developed in this work may be generally applicable to other *bacillus* group strains.

Compared to the CRISPR-Cas9 system, the CRISPR-Cas12f system had a clear comparative advantage. The AsCas12f1 used in our work consisted of 422 amino acid residues, as this was one of the high-activity miniature CRISPR–Cas effectors (Kim et al., 2021; Wu et al., 2021). This characterization work demonstrated that the size of the genome editing plasmid was smaller and higher transformation efficiency was obtained in *B. anthracis* relevant experiments using this effector. At the same time, based on published results, the most efficient PAMs recognized by AsCas12f1 were 5′-TTTR (where R represents A or G) (Karvelis et al., 2020). For *B. anthrcis*, a low-GC-content Gram Positive Bacteria, the distribution of PAMs sequence was thus more general and the spacer screening became easier for many genes. This means that our CRISPR-Cas12f system would be more useful in *B. anthracis* and other low-GC-content bacteria compared to a more traditional SpCas9-based system.

When a one-plasmid-based CRISPR-Cas system was used for two or more gene edits in same parent strain, the rate of editing plasmid curing was shown to be the major constraint and more attention must be paid to overcome this (Wu et al., 2019). According to published studies and our results, some recombinant plasmids, despite including a temperature sensitive origin of replication, are very difficult to cure at the non-permissive temperature in *B. anthracis* and some other *bacillus* species (Hartz et al., 2021). When the plasmids derived from pJOE8999 were used to edit the *B. anthracis* genome, 8–10 passages in antibiotic free medium at the non-permissive temperature were frequently needed to derive transformants without editing plasmids. The period of these experiments were thus lengthened (unpublished data). To solve this issue, separate Cas9 and sgRNA on different plasmids using a two-plasmid system has been a popular method (Wasels et al., 2017; Ao et al., 2018). Here, we also constructed a two-plasmid CRISPR-Cas12f system combined with endonuclease I-*SceI*, a tool enzyme can digest plasmids with specific I-*SceI* sites (TAG​GGA​TAA​CAG​GGT​AAT) *in vivo* (Wang et al., 2018). Two I-*SceI* sites were introduced into the plasmid with target gene-specific sgRNA and homologous arms. After inducing the expression of the endonuclease I-*SceI* on the other plasmid, the plasmid with the sgRNA was digested during growth and passage. The final plasmid curing rate was improved substantially using this method. The interval time was thus shortened for the next target gene editing. Although the curing rate of target plasmids in our work was not as high as some other reports (Jiang et al., 2015), in subsequent research, we will investigate the cause of this and test this method rigorously by selecting more genes of interest as disruption targets and confirm that this is a universal method for gene inactivation in *B. anthracis*. Additionally, other plasmid curing methods will also be studied to optimize and improve this current protocol.

## Data Availability

The original contributions presented in the study are included in the article/Supplementary Material, further inquiries can be directed to the corresponding authors.
