# Supplementary material for: Efficient Genome Editing by a Miniature CRISPR-AsCas12f1 Nuclease in Bacillus anthracis
Source: Front Bioeng Biotechnol. 2022 Jan 14;9:825493. doi: 10.3389/fbioe.2021.825493 (PMC8795892; doi:10.3389/fbioe.2021.825493)
Supplement: Supplementary file 1 [file Table1.DOCX]

Supplementary Material

| **Table S1.** Sequences information of the synthetic DNA |
| --- |
| > The*EcoR*I-*BsrG*I fragment for constructing pJOE-Cas12f1, including *Bacillus anthracis* codon-optimized AsCas12f1 and sgRNA_v1  gaattcttgaccgtgattagagaattgagtaaaatgtacctacgattcgtcggttcagcgacgataagccgagaagtgccaataaaactgttaagtggtttggtaacgctcggtaaggtagccaaaaggctgaaactccgtgcacaaagaccgcacggacgcttcacatatagctcataaacaagggtttgcgagctagcttgtggagtgtgaaccgagaccattggtctcaGGCCGGCATGGTCCCAGCCTCCTCGCTGGCGCCGGCTGGGCAACATGCTTCGGCATGGCGAATGGGACgatctgacagctagcataaccccttggggcctctaaacgggtcttgaggggttttttggatatctaatacgactcactataggggtcgacggccaacgaggcccgggccaataaggcctttctagattaagaaataatcttcatctaaaatatacttcagtcaTTTAATAGATTCTGCAATAATTTTATCAATGTTTGGAATTGCAATGTTACGTGCTGCGTTGTAATCTGCGTTTGCTTCGTAACCACATGCACGACATTTGAAAATTGCTTGACCAATACGGTTACCAGAATCAATGTTACCACATTCAGAACAACGTTGAGATGTGTATTGTGGATCAATTTTAATTACTTTAATACCTGCTTCTTCTGCTTTGTAAATAATTTTTTGTTGTAAATCGTAGTATGTCCAGTTTTGTAAGAAACGAGAACCAATATCACGAATGTTTGTTAAATCTTCCATTTGAATTGTACCACAACCTTCTTTAATTGCCATATCTACAATGTAACGAGAGTAACGATGGTTTGTTGTATCACGGAAGTTTGCAATTTTATCACGTAATTGTTCAATTGGTTTAATACGTTTATCACGACCATGACCACCACGTGCACCACCTGCGTATTTACCTTGACGTAACATAGAAATACGACGAGATTCTACTTGACGACGGAAGTTTTCAATTTCACCACCTTCTAATTTGTAACGTGCTGGTGTATGTTGGAATGCCATGTATACTGCTACTGCTACACCTAAATCAATACCCATAATTTTGTTTAAATCTAATACACGTGTTTGTGGTTCGAAATCGTAAGAAATGTTTAAGTACCATTTGTTTTTACGATCATCATGAATAATTTGAGATGCAGATACTTGGTATTCACCAGATAAAATACGATCCATAATTGTTTTACCTGCACCACGTACAATAATAATTACAGAAATTTTACGTTTTACGTTCATTTCTTGTTTTGCTGGGTTAGATAATAAAGATAAAGATGCAATGTAATCACCATGGTTCATACGGTTTACAGAAATGTTTTCTTTAATTAAATCTAATGGAATATCACGTTTGTAAGATGGAATAGACATATCACCACGTAAAATTTCTTTTTGGTATGCTTTGAAACGATCTGTTGCACGTTTAATTGTTTGAGATAAGTTACCAGAGTTTAAACGGTATGCTTTTGTTTTAATTGTATGGTATGCGTAACCATGTACGTTTGTGTAACCTAAAATATCTTTAGATTTTGGGTATTCACCATGGTTATCTTTGTAATCAGAAGAGAAACCCATCCATTCCCATGCTAATTGTGTTGCTTTGTTTAATGCGAAACGTGTTTCTTGTTGTAATTGACGTAAAATTGTACCAAATTCTTTCCAATCTAAATCTAATGGTTTTACAATTTCGTAACGGTATACTTTAATCATttttgcctcctaacttaagaataagatcttgtctcaactgtataccgaaatcagctcattaaaatcgctttttttaccataggttccggtaataaaggcatttttccctataacaaaaaaagcaaggaataatccctgcttttaataatccaaatgagataaaaatgtcatgacattggTGTACA |
| > bacillus anthracis codon-optimized Mscarlet  CGCTCGAGCCGTCGACGGCTCTAGATTTGGTCTCATGGTATCTAAAGGTGAAGCAGTAATTAAAGAATTTATGCGTTTCAAAGTACATATGGAAGGTTCTATGAACGGTCATGAATTTGAAATTGAAGGTGAAGGTGAAGGTCGTCCATACGAAGGTACACAAACAGCAAAATTAAAAGTAACAAAAGGTGGTCCATTACCATTCTCTTGGGATATTTTATCTCCACAATTCATGTACGGTTCTCGTGCATTCACAAAACATCCAGCAGATATTCCAGATTACTACAAACAATCTTTCCCAGAGGGCTTCAAATGGGAACGTGTAATGAACTTCGAAGATGGTGGTGCAGTAACAGTAACACAAGATACATCTTTAGAAGATGGTACATTAATTTACAAAGTAAAATTACGTGGTACAAACTTCCCACCAGATGGTCCAGTAATGCAAAAAAAAACAATGGGTTGGGAAGCATCTACAGAACGTTTATACCCAGAAGATGGTGTATTAAAAGGTGATATTAAAATGGCATTACGTTTAAAAGATGGTGGTCGTTACTTAGCAGATTTCAAAACAACATACAAAGCAAAAAAACCAGTACAAATGCCAGGTGCATACAACGTAGATCGTAAATTAGATATTACATCTCATAACGAAGATTACACAGTAGTAGAACAATACGAACGTTCTGAAGGTCGTCATTCTACAGGTGGTATGGATGAATTATACAAATAATAGAAACGAATTTTCACAAAAAAATGTGTTTTTCTTTGTTAGACCGGAATTC |
| > bacitracin-inducible promoter  GTCGACTCACACTGGCTCACCTTCGGGTGGGCCTTTCTGCGTTTATATACTAGAGAGAGAATATAAAAAGCCATTACTAATTCACGAGATGATTTCGGTGTGCGTACACCGCCGCCTGCGTCCGGTCACTGACATCCAGCTTTGATAAAATATTCGTAATATGTGTTTTGACTGTTTTAATCGTAATAAACAGTTCCTCGCCTATTTCTTTGTTTGTCTTTCCTTCTGCGATCAGGCAGAGTATTTCGAGCTCCCGTTTTGTAAGCGATTCATGCGGGAGCGCGTTTTCACCTGAGTGGCGCAGCCTGGATAATACTTTTCCCGCCACTTTTGACTCCAGCTTCGGCTCTCCCTTGCTTGCGGCGCGGATGGCATCGGCGATTTCTGCCGCTTTTGAGGTTTTCAACAGATAGCTGAGCGCGCCAGCTTCAATAACCGGGTACACTTTGTCATCATCAATGAAGCTAGTGAGCACAATAATTTTCGGGTCGGAAAGCTCCCGGCAAATTTGCTTTGTAGCTTCAATGCCATCCATGCCCTCCATGACAAGGTCCATTAAAATGACATCAGGCGACAGTTCCACAGCAAGCCGAACACCTTCGCTGCCGTCCGATGCTTCGCCGATGACTTCAATATCGGGCTGCGCCTCCAAAAAAGCCGCGAGCCCCATTCTGACCATTTCATGATCATCAATCAATAATACTCGAATCATATTATATATTTCCTCCTTAGGAATGTAAGATACAAAAATATGTTCAGAGAATGATGCTTCCCTTTGACTCTATGTTAAAACGCTTACAGTCCCTATACAATTGAAAGTAAAGAGGACTTTGGCACATGACTGTGACAATTTTTTAAAAATAAAGCTAGCGGCGTGCACATTGGCCAAAGCAGAAAGGTCCGACCTAATTAAAGAAAGGGAAGCAAGTGTTCATCTGTAAAGGGTTTTAAAACGCCATGCCTCGTGCATGGCGTTTTTTTGTGCCAATGGGTCCGGTGCGAGATACGACTCCGGTCTTATATAAAAATCAATCTCTGATTCGTTTTGCATATCTTCCAACTTGTATAAGATGAAGACAAGGAAAACGACGAAAGGAGGATCTGCATGGTAGAGACC |
| > cumate-inducible promoter, xylose-inducible  GTCGACTCACACTGGCTCACCTTCGGGTGGGCCTTTCTGCGTTTATATACTAGAGAGAGAATATAAAAAGCCATTATTAACGTTTGAATTTTGCGTAACGTTCACGTGCAATTTCTAATGTAGAGTTACGTACACGTTCGAACCTCTCCTTATCTTTTTGCCATAAAGAACGTACTGCTAAACCACGTACAGAGTTGAAAATTAACCATAAAATATCTTCTGCATCATCACGAGATAAACCACGAGATACTAATACACCTAACCACATATCTTCTACTACGAAACGGTTACGTTCTACTGTACGTTGAATACCTTCACGTAATGCTGGATCACGATCTGCTGCTACAATTAAATCTAAAGAAATAGAGAAATCATCATCTAAGAAGAATTCTGCTGCATCATCTAACATTTGTTGAATTACATCATCTTCTGGCTTTAATTTTGCGAGACGTGCACGAGAGCGTTCTGTAATTTGTTCGTATAACCATTCGAATGTTGCTAATAATAATTCTAATTTTGTTGGGAAATGATGAGATTGTGCACCACGAGATACACCTGCTGCACCTGGTACATCTGCAATACGGAAACCTGCGTAACCTTTTTCACGTAATACACCTAATGCTGCTGCAATTAATTTACCTTGTGTTTCCATTGCACGTTCTGCTTGTGTACGACGTTTTGGAGACATGATCACCATTTTTTTTACTAAAGCTTGATCTGCAATTTGAATAATAACCACTCCTTTGTTTATCCACCGAACTAAGTTGGTGTTTTTTGAAGCTTGAATTAGATATTTAAAAGTATCATATCTAATATTATAACTAAATTTTCTAAAAAAAACATTGAAGGAACGGTTATTTGATTATTAATCCGGCTTTTTTATTATTTAGGCAACTGAAACGATTCGGATCCTGTATTACTATTCTATTTTGTCAAAATAATTTTATTGACAACGTCTTATTAACGTTGATATAATTTAAATTTTATTTGACAAAAATGGGCTCGTGTTGTACAATAAATGTAGTAACAAACAGACAATCTGGTCTGTTTGTATTATGATTAACTAATAAGGAGGACAAACATGGTAGAGACC |
| > xylose-inducible from Bacillus megaterium  TGTACAAAGCTTCTAATAACATATAAACAGCCAGTTGCCGTTATGATAGGTGACTGGCTGCATGGGATGAAAAGGTGAGGGTGGAGACAGACATAACACTCTTAATAGAAGAGGGTAATTCTTTCTCTTTTATAGAAAATCAATTAATTGAAAGTAGCTCCTTCATTCTTAAGATCAACGTGATATAGGTTTGCTAACCTTTGCGTTCACTTAACTAACTTATAGGGGTAACACTTAAAAAAGAATCAATAACGATAGAAACCGCTCCTAAAGCAGGTGCATTTTTTCCTAACGAAGAAGGCAATAGTTCACATTTATTGTCTAAATGAGAATGGACTCTAGAAGAAACTTCGTTTTTAATCGTATTTAAAACAATGGGATGAGATTCAATTATATGATTTCTCAAGATAACAGCTTCTATATCAAATGTATTAAGGATATTGGTTAATCCAATTCCGATATAAAAGCCAAAGTTTTGAAGTGCATTTAACATTTCTACATCATTTTTATTTGCGCGTTCCACAATCTCTTTTCGAGAAATATTCTTTTCTTCTTTAGAGAGCGAAGCCAGTAACGCTTTTTCAGAAGCATATAATTCCCAACAGCCTCGATTTCCACAGCTGCATTTGGGTCCATTAAAATCTATCGTCATATGACCCATTTCCCCAGAAAAACCCTGAACACCTTTATACAATTCGTTGTTAATAACAAGTCCAGTTCCAATTCCGATATTAATACTGATGTAAACGATGTTTTCATAGTTTTTTGTCATACCAAATACTTTTTCACCGTATGCTCCTGCATTAGCTTCATTTTCAACAAAAACCGGAACATTAAACTCACTCTCAATTAAAAACTGCAAATCTTTGATATTCCAATTTAAGTTAGGCATGAAAATAATTTGCTGATGACGATCTACAAGGCCTGGAACACAAATTCCTATTCCGACTAGACCATAAGGGGACTCAGGCATATGGGTTACAAAACCATGAATAAGTGCAAATAAAATCTCTTTTACTTCACTAGCGGAAGAACTAGACAAGTCAGAAGTCTTCTCGAGAATAATATTTCCTTCTAAGTCGGTTAGAATTCCGTTAAGATAGTCGACTCCTATATCAATACCAATCGAGTAGCCTGCATTCTTATTAAAAACAAGCATTACAGGTCTTCTGCCGCCTCTAGATTGCCCTGCCCCAATTTCAAAAATAAAATCTTTTTCAAGCAGTGTATTTACTTGAGAGGAGACAGTAGACTTGTTTAATCCTGTAATCTCAGAGAGAGTTGCCCTGGAGACAGGGGAGTTCTTCAAAATTTCATCTAATATTAATTTTTGATTCATTTTTTTTACTAAAGCTTGATCTGCAATTTGAATAATAACCACTCCTTTGTTTATCCACCGAACTAAGTTGGTGTTTTTTGAAGCTTGAATTAGATATTTAAAAGTATCATATCTAATATTATAACTAAATTTTCTAAAAAAAACATTGAAATAAACATTTATTTTGTATATGATGAGATAAAGTTAGTTTATTGGATAAACAAACTAACTCAATTAAGATAGTTGATGGATAAACTTGTTCACTTAAATCAAAGGGGGAAATGACAA |
| > xylose-inducible from Bacillus subtilis  TGTACAAAGCTTCTAATAACATATAAACAGCCAGTTGCCGTTATGATAGGTGACTGGCTGCATGGGATGAAAAGGTGAGGGTGGAGACAGACATAACACTCTTAATAGAAGAGGGTAATTCTTTCTCTTTTATAGAAAATCAATTAATTGAAAGTAGCTCCTTCATTCTTAAGATCAACGTGATATAGGTTTGCTAACCTTTGCGTTCACTTAATTACATTTTAACGATATCTAGAAAATGTTCAATAACAATGGAAGACATTCCTAATGCCGGTGCATTCTTTCCTAAGGAAGATGGTAATAATTCATAGCTATTGCCTAATTGGGGATAAACCCTGGACGACACTTCACTTCTAATTGAATTTAAAACCATAGGATGTGATTCAATTATGCTGTTTCTTAAAATGATGGCTTGTGGATTGAATGTATTTAGAATATTAGTAAGGCCAATTCCTAAATAGAATCCGAAATTCTGTAATGCATTTAAGGTGCCGATATCATTCAGATGGGCGAGGTCTATGATATCTTGATAGGACACTTTTTTCTCTTTAGTCTGAAGAGATTTTAATAAAGCCTTCTCTGAAGCATACAATTCCCAGCAACCTCGGTTTCCGCAACTGCATTTAGGACCATTAAAGTCTATTGTCATATGTCCCATTTCTCCAGAGAATCCGCTTACCCCTCTATATAAATGATTGTTGATAATAACACCGATCCCTATACCTGTACTGATACTAGCATAAATAATGTTATTGTGATTTTTTGCAGCACCAAATACTTTTTCTCCATACGCGCCAGCATTTGCCTCATTTTCAATAAAAACAGGCACATTGAACTTCTCTTGTATGAAAGATTTTAAGTCAATATCTCTCCAGTTGGAGTTCGGAGTGAAAACAATTTTTTGATTTTTATCAATTAGTCCAGGCACGCATATGCCTATACCGATAAGCCCGTACGGAGATTGTGGCATACGCGTAATAAAGTGATGAATCATATCAATTAAAATGTCTTTAGTTATTTCTGGAGAATTCGATTCTAAATGGTGATGTTGATCAAGAATGATCGTTCCTTCAAGGTCTGTTAAAATGCCACTAATATAATCCACACCAACATCTATTCCTATGGAGTATCCTGCCTTTTTATTAAAAACAAGCATGACAGGTCTTCTTCCGCCACTTGATTGTCCTTGACCTATTTCAAATACAAGATTTTCTTTCATCAGCGTGTTTACCTGTGATGAAACAGTTGATTTATTTAATCCAGTCATTTCAGATAATTTTGCTCTTGAAATAGGTGAATTCTTAAGTATTTCTTTTAATAATAACTTTTGATTTACTTTTTTGACAAAGGTTTGATCTGCGATATCCACTTCATCCACTCCATTTGTATAACCTTTAAATTAAGTTAAAATTTTTTGTGTTCAGTATGAGATTTAGTACATAGCGAATCTTACCTTTATTATATCTAATGTGTTCATGAAAAACTAAAAAAAATATTGAAAATACTGATGAGGTTATTTAAGATTAAAATAAGTTAGTTTGTTTGGGCAACAAACTAATGTGCAACTTACTTACAATATGACATAAAATGCATCTGTATTTGAATTTATTTTTAAGGAGGAAATAACAGAGACC |
| >xylR-PxlyA-I-SceI-T7 terminator  TGTACACTAATAACATATAAACAGCCAGTTGCCGTTATGATAGGTGACTGGCTGCATGGGATGAAAAGGTGAGGGTGGAGACAGACATAACACTCTTAATAGAAGAGGGTAATTCTTTCTCTTTTATAGAAAATCAATTAATTGAAAGTAGCTCCTTCATTCTTAAGATCAACGTGATATAGGTTTGCTAACCTTTGCGTTCACTTAATTACATTTTAACGATATCTAGAAAATGTTCAATAACAATGGAAGACATTCCTAATGCCGGTGCATTCTTTCCTAAGGAAGATGGTAATAATTCATAGCTATTGCCTAATTGGGGATAAACCCTGGACGACACTTCACTTCTAATTGAATTTAAAACCATAGGATGTGATTCAATTATGCTGTTTCTTAAAATGATGGCTTGTGGATTGAATGTATTTAGAATATTAGTAAGGCCAATTCCTAAATAGAATCCGAAATTCTGTAATGCATTTAAGGTGCCGATATCATTCAGATGGGCGAGGTCTATGATATCTTGATAGGACACTTTTTTCTCTTTAGTCTGAAGAGATTTTAATAAAGCCTTCTCTGAAGCATACAATTCCCAGCAACCTCGGTTTCCGCAACTGCATTTAGGACCATTAAAGTCTATTGTCATATGTCCCATTTCTCCAGAGAATCCGCTTACCCCTCTATATAAATGATTGTTGATAATAACACCGATCCCTATACCTGTACTGATACTAGCATAAATAATGTTATTGTGATTTTTTGCAGCACCAAATACTTTTTCTCCATACGCGCCAGCATTTGCCTCATTTTCAATAAAAACAGGCACATTGAACTTCTCTTGTATGAAAGATTTTAAGTCAATATCTCTCCAGTTGGAGTTCGGAGTGAAAACAATTTTTTGATTTTTATCAATTAGTCCAGGCACGCATATGCCTATACCGATAAGCCCGTACGGAGATTGTGGCATACGCGTAATAAAGTGATGAATCATATCAATTAAAATGTCTTTAGTTATTTCTGGAGAATTCGATTCTAAATGGTGATGTTGATCAAGAATGATCGTTCCTTCAAGGTCTGTTAAAATGCCACTAATATAATCCACACCAACATCTATTCCTATGGAGTATCCTGCCTTTTTATTAAAAACAAGCATGACAGGTCTTCTTCCGCCACTTGATTGTCCTTGACCTATTTCAAATACAAGATTTTCTTTCATCAGCGTGTTTACCTGTGATGAAACAGTTGATTTATTTAATCCAGTCATTTCAGATAATTTTGCTCTTGAAATAGGTGAATTCTTAAGTATTTCTTTTAATAATAACTTTTGATTTACTTTTTTGACAAAGGTTTGATCTGCGATATCCACTTCATCCACTCCATTTGTATAACCTTTAAATTAAGTTAAAATTTTTTGTGTTCAGTATGAGATTTAGTACATAGCGAATCTTACCTTTATTATATCTAATGTGTTCATGAAAAACTAAAAAAAATATTGAAAATACTGATGAGGTTATTTAAGATTAAAATAAGTTAGTTTGTTTGGGCAACAAACTAATGTGCAACTTACTTACAATATGACATAAAATGCATCTGTATTTGAATTTATTTTTAAGGAGGAAATAACATGCATCAAAAAAACCAGGTAATGAACCTGGGTCCGAACTCTAAACTGCTGAAAGAATACAAATCCCAGCTGATCGAACTGAACATCGAACAGTTCGAAGCAGGTATCGGTCTGATCCTGGGTGATGCTTACATCCGTTCTCGTGATGAAGGTAAAACCTACTGTATGCAGTTCGAGTGGAAAAACAAAGCATACATGGACCACGTATGTCTGCTGTACGATCAGTGGGTACTGTCCCCGCCGCACAAAAAAGAACGTGTTAACCACCTGGGTAACCTGGTAATCACCTGGGGCGCCCAGACTTTCAAACACCAAGCTTTCAACAAACTGGCTAACCTGTTCATCGTTAACAACAAAAAAACCATCCCGAACAACCTGGTTGAAAACTACCTGACCCCGATGTCTCTGGCATACTGGTTCATGGATGATGGTGGTAAATGGGATTACAACAAAAACTCTACCAACAAATCGATCGTACTGAACACCCAGTCTTTCACTTTCGAAGAAGTAGAATACCTGGTTAAGGGTCTGCGTAACAAATTCCAACTGAACTGTTACGTAAAAATCAACAAAAACAAACCGATCATCTACATCGATTCTATGTCTTACCTGATCTTCTACAACCTGATCAAACCGTACCTGATCCCGCAGATGATGTACAAACTGCCGAACACTATCTCCTCCGAAACTTTCCTGAAATAAAGATCTTCCATCGATCGATACCGCTGAGCAATAACTAGCATAACCCCTTGGGGCCTCTAAACGGGTCTTGAGGGGTTTTTTG |
| > The XhoI- *I-Sce*I- sgRNA*-I-Sce*I*-BglII* fragment for constructing pSS-FD  CTCGAGTAGGGATAACAGGGTAATTTGACCGTGATTAGAGAATTGAGTAAAATGTACCTACGATTCGTCGGTTCAGCGACGATAAGCCGAGAAGTGCCAATAAAACTGTTAAGTGGTTTGGTAACGCTCGGTAAGGTAGCCAAAAGGCTGAAACTCCGTGCACAAAGACCGCACGGACGCTTCACATATAGCTCATAAACAAGGGTTTGCGAGCTAGCTTGTGGAGTGTGAACCGAGACCATTGGTCTCAGGCCGGCATGGTCCCAGCCTCCTCGCTGGCGCCGGCTGGGCAACATGCTTCGGCATGGCGAATGGGACGATCTGACAGCTAGCATAACCCCTTGGGGCCTCTAAACGGGTCTTGAGGGGTTTTTTGGATATCTAATACGACTCACTATAGGGGTCGACGGCCAACGAGGCCCGGGCCAATAAGGCCTTTCTAGATAGGGATAACAGGGTAATAGATCT |
